# Systemic Racism Affecting Latinx Population Health During the COVID-19 Pandemic and Beyond: Perspectives of Latinx Community Health Workers and Community-Based Organization Leaders

**DOI:** 10.1089/heq.2023.0193

**Published:** 2023-11-07

**Authors:** Gabriela Plasencia, Rohan Gupta, Kamaria Kaalund, Viviana Martinez-Bianchi, Rosa Gonzalez-Guarda, Andrea Thoumi

**Affiliations:** ^1^ Duke Department of Family Medicine & Community Health, Duke University, Durham, North Carolina, USA.; ^2^ Latinx Advocacy Team & Interdisciplinary Network for COVID-19 (LATIN-19), Durham, North Carolina, USA.; ^3^ Duke-Margolis Center for Health Policy, Duke University, Durham, North Carolina, USA.; ^4^ Duke University, Durham, North Carolina, USA.; ^5^ Duke University School of Nursing, Durham, North Carolina, USA.; ^6^ Duke-Margolis Center for Health Policy, Duke University, Washington, District of Columbia, USA.

**Keywords:** Hispanic or Latino, health equity, determinants of health/population health/socioeconomic causes of health, COVID-19, community-based participatory research, health policy/politics/law/regulation

## Abstract

**Introduction:**

The purpose of this study is to identify forms of systemic racism experienced by Latinx communities in North Carolina during the COVID-19 pandemic as identified by Latinx community health workers (CHWs) and community-based organization (CBO) leaders.

**Methods:**

We conducted three focus groups in July 2022 (*N*=16). We performed qualitative analysis of data using an iterative inductive approach of the original language in Dedoose.

**Results:**

Four central themes emerged: (1) Access to resources for Latinx individuals; (2) Immediate, transitional, and future fears; (3) Benefits of CHWs; and (4) Lessons learned.

**Discussion:**

Institutional and state policies often do not involve community members, such as CHWs and CBO leaders, at the start of the development process, leading to ineffective interventions that perpetuate health disparities and systemic racism.

**Health Equity Implications:**

Community-informed policy recommendations can improve alignment of community and policy priorities to create more effective interventions to address systemic racism and promote health equity.

## Introduction

Marginalized and minoritized populations, such as the Latino/a/x or Hispanic populations (herein referred to as Latinx), experience systemic racism when seeking and receiving health services.^1–3^ The Latinx Advocacy Team and Interdisciplinary Network (LATIN-19) is a community-academic coalition formed in 2020 to reduce health disparities magnified by the COVID-19 pandemic. The coalition's ability to partner with, strengthen relationships, and elevate voices of numerous community-based organizations (CBOs) and community leaders to increase collaboration with decision-makers, such as North Carolina Department of Human Health Services (NCDHHS), Durham County Department of Health (DCDH), and Duke University Health System (DUHS), has resulted in increased community-engaged strategies in policy solutions.^4^

LATIN-19 partners have hosted community events to increase access to COVID-19 testing, vaccination, personal protective equipment (PPE), and food distribution in community-trusted environments with Spanish-speaking volunteers to increase trust and disseminate accurate information. Community engagement, advocacy, and policy efforts led by LATIN-19 helped close the gap in COVID-19 vaccination rates between Latinx and individuals from other races or ethnicities.^5^

The benefits of community health workers (CHWs) and CBOs in improving population health are well documented.^6–8^ For example, CBO community education, outreach, and networking expertise lead to increased hiring and training of local Spanish-speaking CHWs in methods more respectful and acceptable to the Latinx community.^9^ Often, CBOs include CHWs as hourly staff, although CHWs can also provide their expertise to communities independent of a formal organization structure or beyond contracted hours. Together, CHWs and CBOs provide culturally appropriate health information, facilitate system navigation, and build trust with individuals and communities, among other roles.^6,7^ Health systems are increasingly collaborating with CBOs and CHWs to meet needs of high-risk populations.^10,11^ However, CHWs and CBOs are often excluded in policy development or prioritization processes.^12,13^

This study addresses this research-to-implementation gap by engaging with CHWs and CBO leaders affiliated with LATIN-19 to discuss policy and community interventions during the pandemic, how they impacted Latinx communities, and lessons learned for future public health interventions. This study also provides community-informed policy recommendations to increase health equity in Latinx communities. We define health equity as the just opportunity to attain optimal health and well-being by addressing imbalanced processes, policies, and power that create avoidable differences in health outcomes and allocation of resources.^14–17^ The strategies we propose aim to overcome structural barriers to improving health outcomes among Latinx communities while creating trust and communal power.

## Methods

We used a qualitative descriptive design with data collected from three focus groups (*N*=16) in July 2022 via Zoom to address study aims. Participants were recruited through word-of-mouth among research team members. Eligibility criteria were the following: being at least 18 years old; self-identify as Latinx; and self-identify as a CHW or CBO leader. This study was deemed exempt by Duke University's Institutional Review Board as a part of protocol 2022-0294.

Our sample included 7 CBO leaders and 9 CHWs for a total of 16 participants. CBO leaders represented six different CBOs. Each focus group included four to seven participants and was cofacilitated by bilingual and bicultural researchers. Participants were given the option to participate in focus groups in English or Spanish. All participants expressed preference to participate in Spanish. We did not collect sociodemographic data from participants and names were not recorded during focus groups.

Focus group discussions were semistructured with four open-ended questions related to policy and community interventions that improved access to resources during the COVID-19 pandemic, policy or community interventions needed to improve care of Latinx populations, and lessons learned to improve the health of Latinx populations in the future. Participants received $45 after the discussion as compensation for their time.

Focus group discussions were audio-recorded, transcribed in Spanish, and analyzed in the original language. Transcriptions were reviewed by bilingual researchers, and identifying information (such as names or legal status) was redacted. We used inductive thematic analysis, creating codes from constructs that emerged in interviews and applying them to subsequent interviews using a constant comparative approach.^18,19^ Two independent researchers coded each transcript and resolved discrepancies by consensus to enhance credibility of findings.^20^ Dedoose online qualitative data analysis software was used to facilitate team-based coding and data analysis.^21^

## Results

Four themes emerged: (1) unique barriers and facilitators toward resource access for Latinx individuals; (2) immediate, transitional, and future fears related to COVID-19; (3) CHW benefit to Latinx communities; and (4) lessons learned. Below we describe each theme and provide demonstrative quotes that are English translations of the original transcripts in Spanish.

### Access to resources for Latinx individuals

Participants discussed that removing the requirement for proof of identification or insurance decreased fear and improved access to seeking COVID-19-related resources for Latinx individuals. Assurance that all COVID-19-related services, such as testing, vaccination, outpatient treatment, and hospitalization, were available at no cost, regardless of insurance status, also encouraged many Latinx individuals to seek care. Cross-sector collaboration between LATIN-19 and local and state institutions enabled CBOs and CHWs to host many NCDHHS or DUHS-supported events in trusted community locations (e.g., churches and grocery stores), increasing trust and engagement with Latinx individuals who otherwise distrust these institutions due to experiences with structural racism.

Factors cited as decreasing access to COVID-19-related resources included the opportunity cost of seeking care and transportation. Participants stated that the inability to confirm whether Spanish-speaking providers or interpretation services were available at clinical sites was a significant barrier. In addition, clinical sites were often located far from densely Latinx populated areas and high turnover in temporary testing and vaccination sites posed additional barriers to seeking care.

Finally, policies meant to improve access to resources were not always helpful for Latinx populations. For example, many pharmacies required individuals schedule PCR testing or vaccination appointments online, but online platforms were frequently not available in Spanish. Although proof of insurance or identification were not legally required, online platforms and many pharmacies in-person asked for documents to verify patient name and medical record. Furthermore, at-home rapid COVID-19 testing kits neither had consistent instructions nor instructions in Spanish. Therefore, it was difficult for Latinx individuals to understand how to use at-home rapid testing kits correctly.

*The language in many of the pharmacies is not Spanish. So, even though the vaccine is available on every corner, that doesn't mean that the person can go to any corner because the language is not available… So, although it is there, the vaccine, it does not mean that our community can go there.*

### Immediate, transitional, and future fears

#### Immediate fears

Immediate fears included fear of hospitalization, vaccination, symptoms of COVID-19, side effects of COVID-19 vaccination or treatment, and school-related and general asymptomatic transmission of COVID-19. Lack of trustworthy sources of accurate, linguistically and culturally appropriate information was cited as a major contributor to fear of vaccination. Concerns of cost and public charge also contributed to fears of COVID-19 vaccines. Other immediate fears included apprehension toward seeking health-related services, such as vaccines or hospitalizations, which may indicate a broader mistrust of the medical system.

*Many people at first did not get vaccinated out of fear and then because they did not know that this was free and that it was not going to be a public charge, because many people also did not get vaccinated for that reason.*

#### Transitional fears

Transitional fears focused on the evolution of the pandemic before its conclusion. These included fears that local and state institutions will forget about Latinx communities and the contributions of CHWs and CBOs during the pandemic, of return to prepandemic policies, such as need for proof of identification or insurance to obtain resources, and of premature return to normalcy.

*I think the state did very well in giving the opportunity to people like us to work and hopefully they will continue giving funds so that we can continue helping the community, because it is not only with COVID. There is a lot more work to do out there and I know they need it and we are ready.*

#### Future fears

Future fears described post-COVID concerns, including fears of future variants of COVID-19, new viruses such as monkeypox, and of future pandemics. Underlying these fears was focus on a lack of sustainability in funding or other resources for CBOs and CHWs despite ongoing need for outreach related to COVID-19 and other infections.

*We are going to continue working and there are already people who are working without a salary, right? So COVID is not gone and now we are already talking about, well, prevention of other diseases [RSV, influenza] plus monkeypox.*

### Benefits of CHWs

Participants agreed that the role of CHWs during the pandemic was critical to the improvement of health of Latinx communities. Participants highlighted three facets of CHWs that enhanced their impact on community health: (1) CHW competency, (2) CHW coordination skills, and (3) Trust in CHWs.

#### CHW competency

CHWs received trainings in mental health, communication, cardiopulmonary resuscitation, blood pressure screening, and blood glucose screenings. Beyond skillsets related and unrelated to COVID-19, participants described how community members appreciated the quality of care received from CHWs.

*One person told ten and it snowballed from there. We had a waiting list and people were calling us… They said, ‘I love the quality of care they are giving us and I want to bring my friend who lives elsewhere to get vaccinated here’.*

#### CHW coordination skills

CHWs also increased Latinx communities' connection to and navigation of health care systems by providing current information about locations of testing and vaccination centers, especially with Spanish-speaking providers and partnerships with Latinx CBOs and CHWs.

Moreover, CHWs were trained to directly register community members into the electronic medical system and schedule them for testing and vaccination events. Consequentially, Latinx individuals were comfortable providing personal information, such as their address and telephone number, because they were able to discuss the purpose of the information with trusted Spanish-speaking CHWs.

*As time went by and we began to interact with the community, they began to learn how it worked and they began to lose their fear of asking for help when they were sick.*

#### CHW trust

CHWs not only spoke Spanish, but spoke similar forms of Spanish as community members and understood cultural nuances not appreciated by other providers. CHWs also knew how to approach community members with important or sensitive information in ways that felt safe and respectful to Latinx individuals.

*I think giving the right information to our community, that's the main key. Knowing how to approach, knowing how to speak in their language, knowing how to reach out to our people.*

CHWs shared their own experiences with COVID-19 infection or vaccination to reduce misinformation and fears spreading through the community. As a result, community members accepted and identified with CHWs, which increased trust in information and resources CHWs provided.

### Lessons learned

Participants identified four priority areas of lessons learned to improve the health of Latinx populations in the future, including (1) basic necessities and health prevention, (2) information quality and dissemination, (3) funding and sustainability, and (4) the strength of the *Comunidad Latina*. We use the term *Comunidad Latina*, which was selected as the preferred term used by LATIN-19 community members.^22^

#### Basic necessities and health prevention

Inclusion of basic necessities, such as culturally appropriate food items, PPE, at-home rapid testing kits, and resources not directly related to COVID-19, such as help signing up for Supplemental Nutrition Assistance Program benefits, was critical to improving care of Latinx communities during the pandemic. Participants reported that provision of basic necessities related and unrelated to COVID helped keep families at home, decreasing transmission risk and increasing institutional and CBO trust.

#### Information quality and dissemination

Participants highlighted the importance of timely linguistically appropriate printed and video materials. Delays in information dissemination often occurred for Spanish-speaking communities because handouts were initially available in English and only later in Spanish. Relatable and community-reviewed sources of information were also critical in effective information dissemination.

*I think that education and prevention is the most important thing, apart from the fact that it is done by a person who identifies as Latino. Because it is not the same as a white doctor coming in and saying, ‘Oh, you do this’.*

Participants provided an example of a public announcement in Spanish that directly translated in English to “cover your cough,” instead of “cover your mouth when you cough.” Participants stated that this translation likely resulted from lack of engagement with Spanish-speaking community members during development of the announcement, a common pattern with translated messaging during the pandemic. In addition, participants stated that messaging from trusted community-recognized leaders, such as the Latinx Spanish-speaking physicians associated with LATIN-19, had significantly greater impact on community health prevention and care-seeking behaviors than messaging from leaders unfamiliar to Latinx communities. Finally, repeating information in memorable, digestible ways, such as the 3 M's (the Spanish-language version of 3W's: “Wash your hands, wear a mask, and wait 6 feet”) was effective, especially if taught to children and adults alike, because children often influenced adults to change their behavior.

#### Funding and sustainability

Given the wealth of information, resources, and skills acquired by CBOs and CHWs during the pandemic, sustainability and funding were significant concerns for participants. Participants noted that community-based interventions improved testing and vaccination rates among marginalized subgroups in the population, such as farmworkers, factory workers, and undocumented individuals. However, funding was not necessarily distributed to CBOs based on strengths or prior expertise. Moreover, frequent changes in funding sources for community events throughout the pandemic led to increased turnover of CBOs and CHWs and impeded sustainable relationship building. Participants stated that this was likely because decisions were made by leaders from outside Latinx communities with little engagement with existing CBOs and CHWs in the process. These situations led to competition between CBOs, fractioning connections within Latinx communities, and decreasing their collective power to inform meaningful change.

#### Comunidad Latina

Despite the lack of trustworthy Spanish-language information or connections to the health care system, participants discussed ways in which the *Comunidad Latina* supported itself, demonstrating its resilience and determination. Participants discussed how CBOs and CHWs often worked without pay when other organizations and institutions closed because they were acutely aware of the dire need for help and the skyrocketing death rates within Latinx communities.

The partnership that eventually formed between CBOs, CHWs, LATIN-19, NCDHHS, DUHS, and others strengthened a network of support for the Latinx community that increased the bidirectional flow of information between CHWs and CBOs at the frontlines and state decision-makers. Participants stated Latinx CBOs and CHWs contributed significantly to public health efforts at local and state levels during the pandemic. However, the lack of visibility of these efforts outside of those directly involved posed a significant impediment to the sustainability and strength of this network.

*They could not have done the job without us [CBOs & CHWs]. Definitely. So, we have the capacity and we need credit, the credit that we can do it.*

## Discussion

We examined Latinx CHW and CBO leaders' perceptions of policy and community interventions and key lessons learned during the pandemic to improve the health of Latinx communities in the future. Overall, the experiences shared demonstrate instances in which lack of community-engagement led to ineffective and potentially harmful interventions. The findings from this study provide needed engagement strategies with Latinx CHW and CBO leaders that can be leveraged to inform equity-centered policies and systems-level changes to promote more equitable health systems.

One particular strategy for community engagement and health equity highlighted during the pandemic and in our study is the inclusion of CHWs, especially for Latinx and Black communities.^23^ CHWs offer an evidence-based solution to improve health disparities in diagnosis and treatment of chronic conditions, such as diabetes, hypertension, and obesity among marginalized and minoritized populations.^6^ With community-focused recruitment and support, CHWs can be transformative to health care delivery systems, particularly for Latinx communities.^8^

In addition, the lessons learned demonstrate several actionable policy recommendations that will continue gains while mitigating structural racism perpetuated by interventions developed during the pandemic. These include (1) cocreating educational materials in other languages when created in English, (2) integrating Latinx CBO leaders, CHWs, and others directly connected to the community in discussion of policies impacting Latinx populations for consideration of possible unintended consequences, (3) prioritizing activities valued by Latinx communities, such as community-based events, to maintain or regain the community's trust, and (4) equitably distributing funding for CBOs among experienced organizations to foster collaboration.

Our study had some limitations. These findings are based on the perceptions of few Latinx CHWs and CBO leaders, and therefore may not be broadly generalizable. We did not collect sociodemographic information and, thus, cannot evaluate differences in perceptions based on immigration status, education level, and so on. We only included Spanish-speaking individuals, therefore, do not have insights about the experiences of other Latinx populations who do not speak Spanish. Moreover, there is limited language around institutional and systemic racism in many Latinx communities even though it manifests in a myriad of ways.

## Health Equity Implications

The end of the Public Health Emergency Declaration has reversed many policies that our participants credited with improving access to resources and community trust in local and state institutions, such as no cost COVID-19-related testing, vaccination, and treatment. CBO leaders and CHWs alike noted the marked decrease in community events after the transition to “normalcy.” Such changes affirm several of the transitional and future fears discussed by focus group participants, dismantling many of the advances in health equity by impairing institutional trust and engagement accomplished during the pandemic.

Implications of the Public Health Emergency unwinding highlight the importance of reaching community members utilizing strategies outside of the traditional clinic and hospital structure, whether through CHWs, temporary clinical sites in trusted community locations, or mobile van units. As noted by the CHWs, COVID-19 testing and vaccination was often the first touchpoint Latinx individuals had with DUHS. This directly demonstrates that more community outreach and nontraditional health care delivery methods are needed to improve health equity for Latinx populations related to COVID-19 and beyond.

In addition, as demonstrated by our study, interventions designed without community involvement can misalign resources with needs and perpetuate forms of systemic racism. Therefore, including CHWs and CBO leaders along with policymakers in policy-making processes would improve visibility around the invaluable work these organizations and individuals contribute toward achieving health equity.

Collaboration of CBOs and CHWs with community-academic partnerships can also improve public health efforts, community interventions, and trust in health care systems, especially during the pandemic.^24^ The multisector nature of LATIN-19 has allowed the coalition to inform advocacy, research, and policy recommendations to improve the health of Latinx communities.^4,5^ Collaboration, trust, and communal power developed by such multisector coalitions has the potential to improve health equity for marginalized and minoritized populations beyond the efforts of individual interventions or organizations.^25^ Therefore, efforts to develop, evaluate, and sustain these coalitions are in the financial and moral interest of health care systems, public health departments, and local and state policymakers.

## Abbreviations Used

CBO: community-based organization
CHWs: community health workers
DCDH: Durham County Department of Health
DUHS: Duke University Health System
LATIN-19: Latinx Advocacy Teamwork & Interdisciplinary Network for COVID-19
NCDHHS: North Carolina Department of Human Health Services
PPE: personal protective equipment
